# An Early Presentation of Spontaneous Intestinal Perforation in a Very Low Birth Weight Neonate: A Case Report

**DOI:** 10.7759/cureus.42285

**Published:** 2023-07-22

**Authors:** Neelam Harsha, Suresh Babu Mendu, Avinash Santhosh, Rakesh Kotha, Madireddy Alimelu

**Affiliations:** 1 Department of Neonatology, Niloufer Hospital, Hyderabad, IND; 2 Department of Pediatrics, Government Medical College, Siddipet, IND; 3 Department of Pediatrics Intensive Care, Osmania Medical College, Hyderabad, IND; 4 Department of Neonatology, Osmania Medical College, Hyderabad, IND

**Keywords:** spontaneous intestinal perforation, necrotizing enterocolitis, preterm neonate, lower gastrointestinal tract perforation, general pediatric surgery

## Abstract

Pneumoperitoneum is typically caused by breached hollow viscera and necessitates surgical intervention. This may have various etiologies, including spontaneous, necrotizing enterocolitis (NEC), and obstruction. In these cases, spontaneous intestinal perforation (SIP) is a unique clinical entity with a better outcome than newborns with NEC-related intestinal perforation. Here, we present a rare case of SIP manifested in the form of pneumoperitoneum in the first eight hours of life, emphasizing the importance of differentiation between NEC and SIP, as each condition has variable treatment options and outcome considerations.

## Introduction

Gastrointestinal perforation (GIP) in neonates is a major challenge, and mortality can be high [1]. Necrotizing enterocolitis (NEC) is the most common cause of neonatal GIP worldwide, ahead of other causes such as mechanical obstruction and spontaneous bowel perforation [1]. Spontaneous intestinal perforation (SIP) in neonates, also known as focal bowel perforation, is a single bowel perforation that typically occurs in the terminal ileum [2]. The incidence of SIP has been reported to be 1%-2% in very low birth weight (VLBW) babies [3] and 5%-8% in extremely low birth weight (ELBW) babies [4]. In addition, the incidence of SIP has been documented to increase with decreasing gestational age, and the median age of onset is seven days with a range of 0-15 days [5]. Here, we present a rare case of SIP in a preterm infant that occurred as early as eight hours after birth, prompting surgical treatment with intraoperative and postoperative findings.

## Case presentation

A male baby was born to a primi mother via a spontaneous vaginal delivery at 30+2 weeks of gestation with a history of preterm premature rupture of membranes (pPROM) for 20 hours. The mother received only one dose of steroids four hours before the delivery. The baby’s birth weight was 1240 gm, and the APGAR scores were 8 and 9 at 1 and 5 minutes, respectively. The provisional diagnosis of a premature baby with respiratory distress syndrome was kept, and the baby was admitted to the neonatal intensive care unit with respiratory support of noninvasive ventilation (NIV) via a RAM cannula. Given the history of pPROM, the baby was empirically started on ciprofloxacin and gentamycin as they were first-line antibiotics during that period. Parenteral nutrition was also initiated. As respiratory distress persisted with a Silverman Anderson score of 5/10, on NIV at 18/6 and FiO_2_ at 60%, surfactant was administered uneventfully using the less invasive surfactant administration technique. The plan was to start minimal enteral nutrition subsequently; however, abdominal distension was noted at six hours of life. There were no documented Doppler changes antenatally. The baby did not experience episodes of vomiting, the OG tube was in situ, and no aspirates were noted. The baby passed a small quantity of meconium immediately after birth.

**Figure 1:**
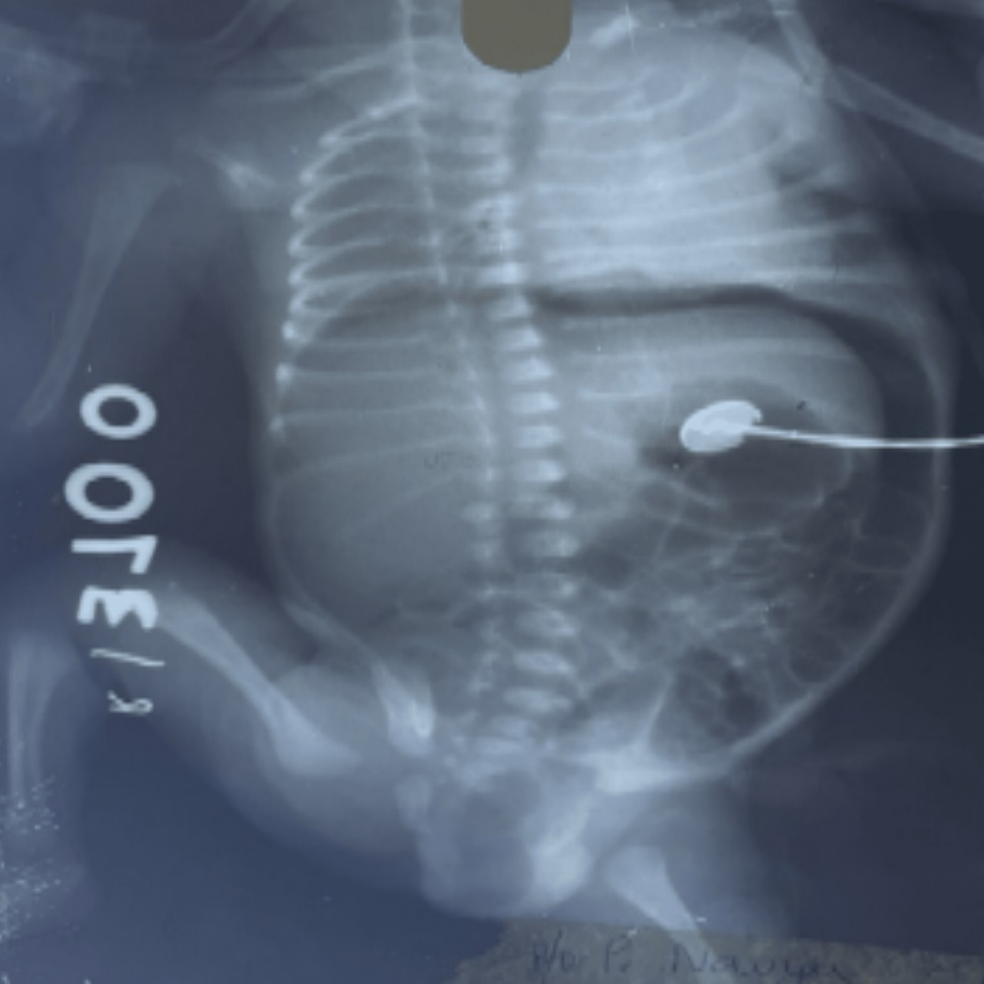
Abdominal radiograph in supine view showing pneumoperitoneum

Based on the pPROM history, the possibility of sepsis/septic ileus or NEC was considered. Thus, the baby was kept nil per oral (NPO). Subsequently, an abdominal radiograph was obtained, showing gas under the diaphragm suggestive of pneumoperitoneum (Figure 1), with no other features of NEC. The working diagnosis was SIP. 

**Figure 2:**
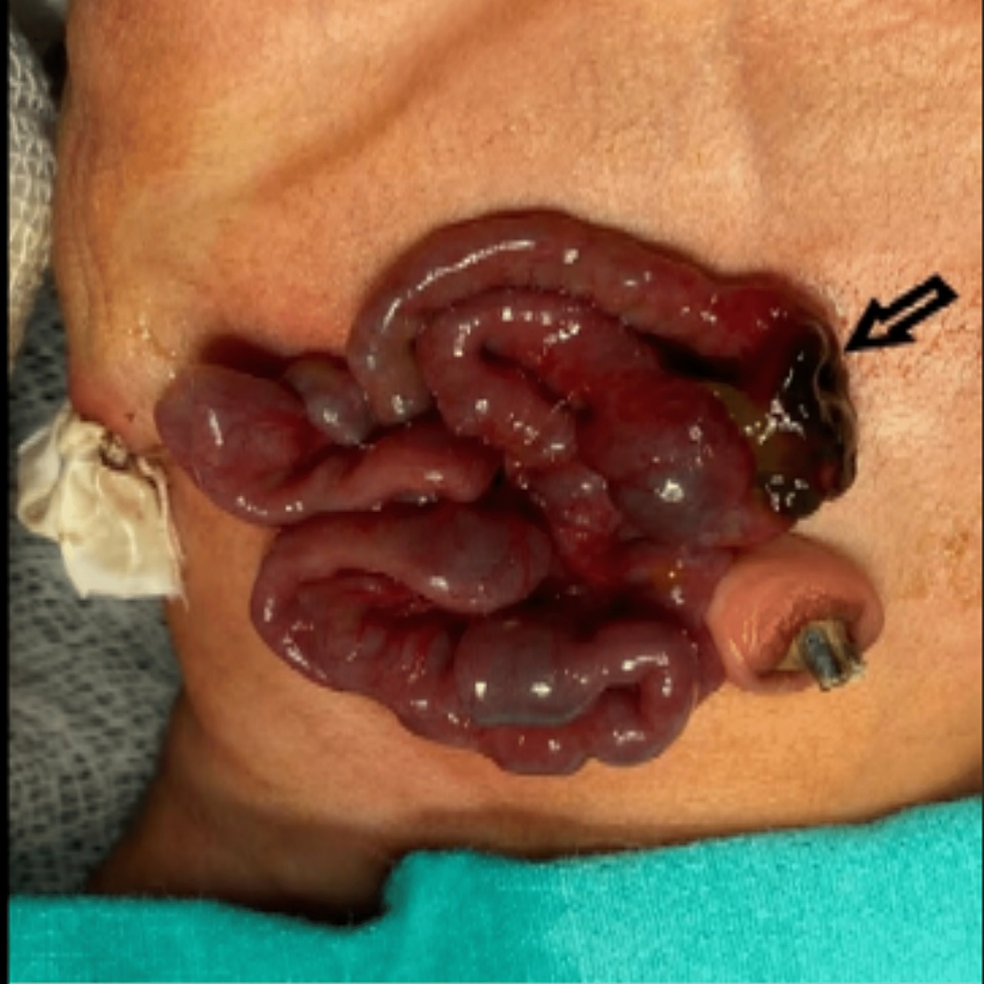
Intraoperative finding of ileal perforation (arrow mark)

A pediatric surgeon’s opinion was sought, and a glove drain was placed when a gush of air was noted. For further management, the baby was taken for an extended laparotomy. The intraoperative findings included 1 x 1.5 cm perforation in the ileum, 40 cm from the ileocecal junction, with minimal meconium collection in the pelvis (Figure 2).

**Figure 3:**
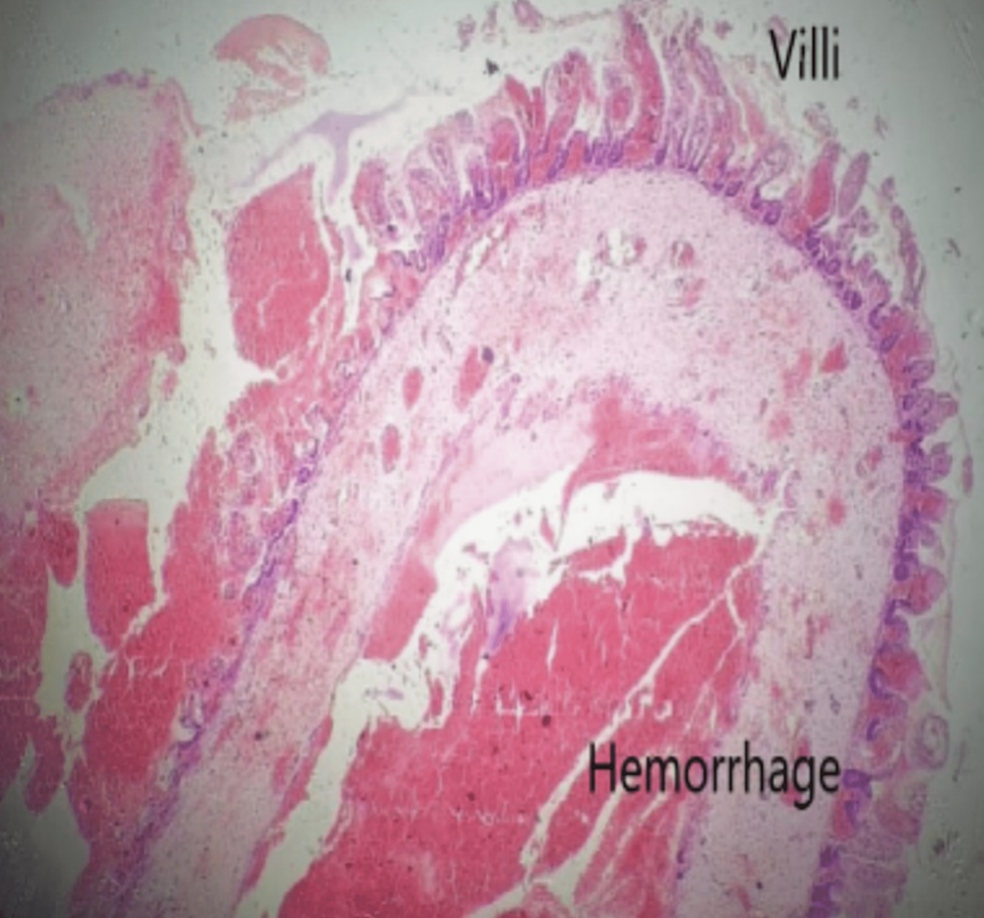
Histopathological slide showing inflammation and haemorrhage

Three centimeters of intestine was resected, and end-to-end ileoileal anastomosis was done without tension. Histopathology revealed transmural inflammation, vascular congestion, areas of hemorrhage, ischemic necrosis, and the presence of mature ganglion cells in the metric plexus (Figure 3).

The baby had an uneventful postoperative recovery. The incision wound was dressed regularly, and enteric feeding was initiated minimally one week after surgery and gradually increased. By day 15 of life, the baby reached full feeds, was hemodynamically stable, and was gaining weight.

## Discussion

SIP of the small bowel is a condition that occurs in premature infants, is not associated with the clinical and pathological features of NEC, and is not due to obstructive phenomena such as Hirschsprung’s disease [6]. Therefore, it is a distinct clinical entity said to result in a better outcome than neonates with intestinal perforation secondary to NEC [7].

Perforation is likely the clinical consequence of multiple pathological processes contributed by both congenital and acquired factors. It is associated with prematurity, low birth weight, asphyxia at birth or placement of an umbilical artery catheter, ischemia, and exposure to indomethacin or dexamethasone [8]. Of these factors, the present baby was premature and of VLBW status. The incidence of SIP increases with decreasing gestational age, and the median age of onset is seven days, with a range of 0-15 days [8]. That implies that SIP usually presents earlier in life, with varying risk factors according to the time of presentation [8]. In comparison, NEC occurs later, usually after the introduction of feeds. In our case, the NEC-like clinical picture occurred as early as day 1 of life, before the introduction of feeds, thus implying a possibility of SIP.

Abdominal radiographs that demonstrate pneumoperitoneum support the diagnosis of SIP, as seen in our case (Figure 1). Sometimes, a gasless abdomen can also be seen. Radiological findings in NEC may include portal gas or pneumatosis intestinalis, which were not seen in our case.

SIP management involves both medical and surgical management. Medical management consists of unwinding the gastrointestinal tract for 7-14 days, providing circulatory support, administering antibiotics, and using respirator support if necessary. Similarly, in our case, NIV was used to provide respiratory support, an orogastric tube was inserted, and the baby was kept NPO. The best surgical treatment for SIP is debatable. The typical surgical method is exploratory laparotomy with bowel resection, but primary peritoneal drainage is another approach. A prospective randomized trial conducted by Moss et al. in the case of NEC or SIP perforation found no difference in the results between the two techniques [9]. We did glove draining at our institute to prevent additional distension and decompensation, followed by definitive surgery.

The infant intestinal perforation mortality rate remains high, ranging from 40% to 70% [10]. According to Prgomet et al., infant intestinal perforation has a 31% death rate [11]. According to a study conducted by Hyginus et al., NEC, preterm status, low birth weight, multiple perforations, and delays in diagnosing perforations, all increased infant GIP mortality. Many cases of SIP were managed by glove drain alone, and SIP has a better prognosis than NEC, with a recovery rate of 70%-100% [1]. Thus, after adjusting for confounding factors, studies have shown that SIP appears to have a lower mortality rate than NEC, though they have a similar risk for neurodevelopmental impairment [12]. Early identification and diagnosis helped prompt intervention leading to survival in our case.

## Conclusions

Thus, the authors highlight the importance of distinguishing between SIP and NEC-related perforation. A detailed evaluation might help identify a pattern for earlier recognition and intervention for this condition. Any perforation without starting feed in neonates should be suspected as SIP. SIP usually has a good prognosis, and post-surgical complications are less common.
